# Cytotoxicity of Plant-Mediated Synthesis of Metallic Nanoparticles: A Systematic Review

**DOI:** 10.3390/ijms19061725

**Published:** 2018-06-11

**Authors:** Nurul Akma Hanan, Hock Ing Chiu, Muggundha Raoov Ramachandran, Wai Hau Tung, Nur Nadhirah Mohamad Zain, Noorfatimah Yahaya, Vuanghao Lim

**Affiliations:** 1 Active Pharmaceutical Ingredient (API) Section, Centre of Product Registration, National Pharmaceutical Regulatory Agency (NPRA), Lot 36, Jalan Universiti, 46200 Petaling Jaya, Malaysia; nurulakma282@gmail.com; 2 Integrative Medicine Cluster, Advanced Medical and Dental Institute, Universiti Sains Malaysia, 13200 Bertam, Penang, Malaysia; hocking6179@gmail.com (H.I.C.); nurnadhirah@usm.my (N.N.M.Z.); noorfatimah@usm.my (N.Y.); 3 Department of Chemistry, Faculty of Science, Universiti Malaya, Kuala Lumpur 50603, Malaysia; muggundha@um.edu.my; 4 School of Pharmacy, University of Nottingham Malaysia Campus, Jalan Broga, Semenyih 43500, Malaysia; Tung.WaiHau@nottingham.edu.my

**Keywords:** cytotoxicity, plant, synthesis, metallic nanoparticles

## Abstract

In the field of medicine, nanomaterials, especially those derived using the green method, offer promise as anti-cancer agents and drug carriers. However, the biosafety of metallic nanoparticles used as anti-cancer agents remains a concern. The goal of this systematic review was to compare the cytotoxicity of different plant-mediated syntheses of metallic nanoparticles based on their potency, therapeutic index, and cancer cell type susceptibility in the hopes of identifying the most promising anti-cancer agents. A literature search of electronic databases including Science Direct, PubMed, Springer Link, Google Scholar, and ResearchGate, was conducted to obtain research articles. Keywords such as biosynthesis, plant synthesis, plant-mediated, metallic nanoparticle, cytotoxicity, and anticancer were used in the literature search. All types of research materials that met the inclusion criteria were included in the study regardless of whether the results were positive, negative, or null. The therapeutic index was used as a safety measure for the studied compound of interest. Data from 76 selected articles were extracted and synthesised. Seventy-two studies reported that the cytotoxicity of plant-mediated synthesis of metallic nanoparticles was time and/or dose-dependent. Biosynthesised silver nanoparticles demonstrated higher cytotoxicity potency compared to gold nanoparticles synthesised by the same plants (*Plumbago zeylanica*, *Commelina nudiflora*, and *Cassia auriculata*) irrespective of the cancer cell type tested. This review also identified a correlation between the nanoparticle size and morphology with the potency of cytotoxicity. Cytotoxicity was found to be inversely proportional to nanoparticle size. The plant-mediated syntheses of metallic nanoparticles were predominantly spherical or quasi-spherical, with the median lethal dose of 1–20 µg/mL. Nanoparticles with other shapes (triangular, hexagonal, and rods) were less potent. Metallic nanoparticles synthesised by *Abutilon inducum*, *Butea monosperma*, *Gossypium hirsutum*, *Indoneesiella echioides*, and *Melia azedarach* were acceptably safe as anti-cancer agents, as they had a therapeutic index of >2.0 when tested on both cancer cells and normal human cells. Most plant-mediated syntheses of metallic nanoparticles were found to be cytotoxic, although some were non-cytotoxic. The results from this study suggest a focus on a selected list of potential anti-cancer agents for further investigations of their pharmacodynamic/toxicodynamic and pharmacokinetic/toxicokinetic actions with the goal of reducing the Global Burden of Diseases and the second leading cause of mortality.

## 1. Introduction

Nanotechnology has been embraced by industrial sectors due to the tremendous number of potential applications of nanoparticles and nanomaterials in diverse fields including engineering, telecommunications, advertising, electronics, textiles, space and defence, cosmetics, and medicine. In medicine, nanotechnology is being used to develop new antibacterial and anti-cancer agents in the hopes of devising better treatments and strategies for combating cancer. Cancer is a major health problem worldwide and it is listed as one of the Non-Communicable Diseases in the Global Burden of Diseases. It is the second leading cause of mortality after cardiovascular disease, accounting for the deaths of 8.8 million people worldwide in 2015 [1].

The use of nanoparticles as a potential strategy for combating cancer has been extensively studied since the early 2000s. Recently, researchers have been developing anti-cancer agents using metallic nanoparticles synthesised via various methods, including mechanical attrition, laser ablation, photo reduction, chemical electrolysis, and synthesis by organisms such as bacteria and plants. Biologically synthesised (that is, green) metallic nanoparticles are favoured because chemical and physical methods have many drawbacks, including the use of toxic solvents, generation of hazardous by-products, and high energy consumption [2]. Plant-mediated nanoparticle synthesis is preferred over other techniques because biologically active plant compounds can be exploited as key resources during green synthesis [3]. Additionally, this approach does not involve complicated processes of intracellular synthesis, multiple purifications, and the maintenance of microbial cells [4].

The plant-mediated synthesis of metallic nanoparticles has become increasingly recognised as a mean to produce cytotoxic agents to fight various cancers [5,6,7,8,9,10,11,12]. However, the safety of these synthesised cytotoxic nanomaterials as a treatment remains a huge concern. A potent anti-cancer agent may kill not only cancer cells but also normal healthy cells when used as a treatment. An ideal drug should be selective, specific to the target site, potent (<100 mg/day), safe, effective, and have minimal food/drug interactions, convenient dosing frequency, and no requirement for blood level monitoring [13]. Therefore, researchers aim to engineer an anti-cancer agent that has a wide therapeutic index and is stable, specific towards the target site, biocompatible, biodegradable with minimal side effects, reasonably simple to reproduce, and cost-effective.

Hence, the goals of this systematic review were to compare the cytotoxicity potency of the plant-mediated synthesis of metallic nanoparticles based on the results of recently published studies (2006 to 2017); to identify the plant-mediated synthesis of metallic nanoparticles with the most promise as anti-cancer agents based on their potency, cancer cell type susceptibility, and therapeutic index; and to correlate nanoparticles size and morphology with the potency of cytotoxicity.

## 2. Results

The Science Direct database was searched on 11 November 2016, and the process yielded 319 articles. The search of the PubMed database was conducted on 30 November 2016 and yielded 17 articles. On 8 December 2017, the Springer Link database was searched, which produced 291 articles. The Google Scholar search engine was queried on 16 December 2017, resulting in the retrieval of 160 articles. Lastly, 9 articles were retrieved from ResearchGate on 17 December 2017. After the articles were imported, the results from the databases were merged, with a total of 796 articles retrieved. Nineteen duplicates were removed. Abstract screening of the remaining 777 articles identified 671 articles that were unrelated to the research question. Of the remaining 106 articles, 88 met the inclusion criteria and 18 studies were excluded.

The remaining 88 articles were critically appraised using the criteria described in Section 4. Twelve articles were excluded after thorough appraisal. Seven articles were excluded due to insufficiently described methodology (for example, duration of exposure not stated clearly for an in vitro study), one study was excluded because the dosing of plant-mediated synthesis of metallic nanoparticles was not specified, and one study was excluded because the experiment was not conducted at body temperature. One other article was excluded because the data for the treatment outcomes were not available. Finally, two articles were excluded because the outcomes for each treatment (such as 24, 48, and 72 h) were not reported separately. Thus, 76 studies were included in the qualitative synthesis. Of these, only 18 studies were used for the quantitative synthesis and analysis.

In vitro data were available in 75 studies, whereas in vivo results were only present in 1 study. Twenty-one studies focused on plant-mediated gold nanoparticles alone, 52 studies focused on plant-mediated silver nanoparticles alone, and 3 articles focused on both metals simultaneously.

### 2.1. In Vitro Studies

Table 1 provides the data about the cytotoxicity against cancer cells (in vitro) for each plant used in the synthesising of metallic nanoparticles. Most of the plant-mediated syntheses of metallic nanoparticles (67 studies) showed cytotoxicity to cancer cells such as human epithelial lung carcinoma (A549), hepatocellular carcinoma (Hep3B), gastric adenocarcinoma (AGS), human colon carcinoma (HCT-116), human breast adenocarcinoma (MCF-7), human adenocarcinoma mammary gland (MDA-MB), human epithelioid cervix carcinoma (HeLa), human promyelocytic leukaemia (HL-60), and glioblastoma (U87) cells.

The table shows that the cytotoxicity of the synthesised nanoparticles is time and/or dose-dependent. However, gold nanoparticles synthesised from *Genipa Americana* fruit were non-cytotoxic. Although the gold nanoparticles synthesised from the *Dysosma pleiantha* rhizome showed no cytotoxicity to human fibrosarcoma (HT1080) cells over the tested durations, the study highlighted their anti-metastatic property by cell migration inhibition via the Rac1-mediated actin polymerisation pathway [68]. Overall, the silver nanoparticles had a higher cytotoxicity potency than gold nanoparticles when the same plants (*Plumbago zeylanica*, *Commelina nudiflora*, and *Cassia auriculata*) were used for the synthesis, irrespective of the cancer cell type tested [5,14,71].

The most potent cytotoxic plant-mediated synthesis of the metallic nanoparticles was the silver nanoparticles synthesised by *Oleo europaea* (Olive) leaves: an LD_50_ of only 24 ng/mL over 24 h was needed to kill breast cancer (MCF-7) cells. Silver nanoparticles derived from *Cassia auriculata* leaves were the most potent in eradicating lung cancer (A549) cells: 100% cell death occurred in all tested concentrations, with 10 µg/mL being the minimum tested dose. Gold nanoparticles derived from the leaves of the same plant also led to the complete cancer cell death at a dose of 30 µg/mL. *Cajanus cajan* (Pigeon pea)-mediated gold nanoparticles had an LD_50_ of 6 µg/mL over the course of 24 h on liver cancer (HepG2) cells. *Iresine herbstii*-mediated silver nanoparticles were the most effective at killing cervical cancer (HeLa) cells over 3 h with an LD_50_ of 51 µg/mL.

Twenty-five of the selected studies tested the cytotoxicity of plant-mediated syntheses of metallic nanoparticles on normal cells (Table 2), which included human lymphocytes, peripheral blood mononuclear cells (PBMC), human breast epithelial cells (HBL100), human keratinocytes (HaCaT), human foetal lung cells (WI-38), African green monkey kidney cells (Vero), mouse fibroblasts (L929), and other cells.

Twenty-four of the studies of plant-mediated synthesis of metallic nanoparticles reported time- and/or dose-dependent cytotoxicity against normal healthy cells. However, four studies reported a contradictory effect. Gold nanoparticles synthesised from *Mangifera indica* (Mango) peel and *Genipa Americana* fruit resulted in <20% normal cell death for the entire tested dose over the duration of exposure. Silver nanoparticles biosynthesised using the *Oleo europaea* (Olive) leaf and *Nyctanthes arbortristis* (Night Jasmine) flower also caused <20% normal cell death for the entire tested doses over the duration of exposure. Metallic nanoparticles derived from the Mango peel, *Genipa Americana* fruit, and Night Jasmine flower showed no cytotoxic activity, which suggests that the use of these plants as drug-delivering carriers in future medicinal products would be safe. Table 1 shows that the *Oleo europaea*-derived silver nanoparticles have good potency against breast cancer (MCF7) cells, but they do not eradicate normal healthy cells (Table 2). This indicates that the cytotoxicity of these silver nanoparticles was selective towards the tested cancer cells.

### 2.2. In Vivo Studies

The preliminary search results included two studies in which the in vivo cytotoxicity of plant-mediated synthesis of metallic nanoparticles was studied. They were conducted using Dalton’s ascites lymphoma (DAL) mouse model and adult zebrafish (*Danio rerio*). However, only the zebrafish study met all of the required criteria for a quality assessment of the study. Table 3 provides the study description and mortality and cytotoxicity results for the in vivo plant-mediated synthesis of metallic nanoparticles. Silver nanoparticles were synthesised using *Malva crispa* leaves. The median lethal dose was 142.2 ng/mL, and 100% mortality occurred at 331.8 and 284.4 ng/mL at 48 and 96 h, respectively. The cytotoxicity of the plant-mediated synthesis of metallic nanoparticles was prominent as the cell membranes of adult zebrafish gill tissues were damaged and gill cells were destroyed.

### 2.3. Safety of Plant-Mediated Synthesis of Metallic Nanoparticles

A high therapeutic index indicates a larger safety margin. In human breast cancer (MCF-7) cells, *Cassia fistula* and *Melia dubia*-derived silver nanoparticles appeared to be the safest, with therapeutic index values of 9.23 and 16.03 after exposure for 24 and 48 h, respectively. However, both studies compared the cytotoxicity potency against animal cells (African green monkey kidney (Vero) cells) instead of normal human cells. In MCF-7 cells, the silver nanoparticles synthesised from *Annona squamosa* and *Oleo europaea* (Olive) had therapeutic index values of <2 when their cytotoxicity was compared with normal human cells.

In human cervical cancer (HeLa) cells, all plant-mediated synthesis of metallic nanoparticles had a therapeutic index of ≤ 2.5. *Melia azedarach*-derived silver nanoparticles had the highest therapeutic index compared to other plants. In addition, when the therapeutic index of cytotoxicity of these nanoparticles was compared to a cytotoxic drug, 5-fluorouracil (5-FU), on the same cancer cells and normal cells, the plant-synthesised metallic nanoparticles were safer than 5-FU [62].

In human colon cancer (HT29) cells, *Abutilon indicum*-derived gold nanoparticles had a therapeutic index of 4.76 and 5 after exposure of 24 and 48 h, respectively. Both *Indoneesiella echioides*-derived and *Gossypium hirsutum* (Cotton)-derived silver nanoparticles were equally safe, having a therapeutic index of 2 when tested on human epithelial lung carcinoma (A549) cells and human breast epithelial (HBL100) cells over 48 h of exposure. *Butea monosperma*-derived silver nanoparticles had a therapeutic index of 3.77 when tested over 24 h on both human myeloid leukaemia (KG-1A) cells and human peripheral blood mononuclear cells (PBMC). In summary, the metallic nanoparticles synthesised from *Abutilon inducum*, *Butea monosperma*, *Gossypium hirsutum*, *Indoneesiella echioides*, and *Melia azedarach* were acceptably safe, as their therapeutic index values were ≥2.0 when tested on both cancer cells and normal human cells.

### 2.4. Size and Cytotoxicity

**Figure 1:**
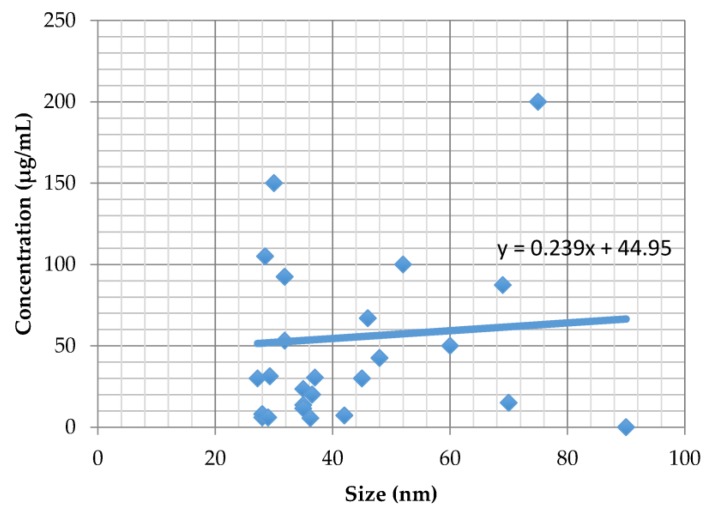
The correlation between the average size of the plant-mediated syntheses of metallic nanoparticles and cytotoxicity at 24 h of exposure. 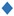 Plant metallic nanoparticles; 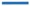 Trendline.

**Figure 2:**
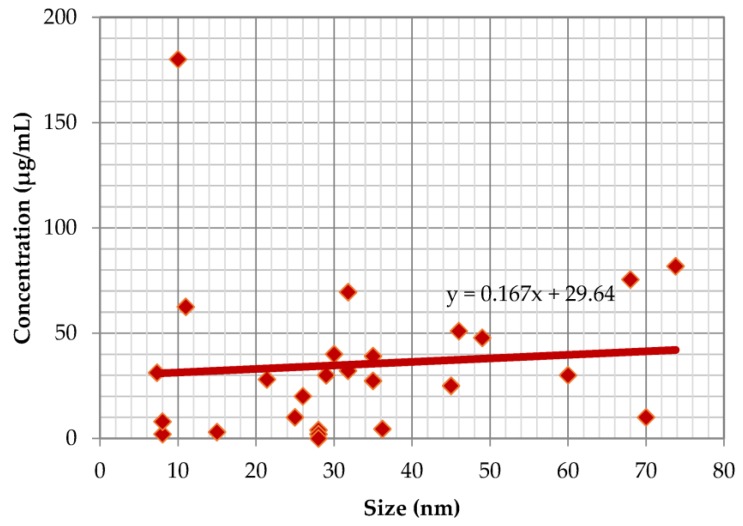
The correlation between the average size of the plant-mediated syntheses of metallic nanoparticles and cytotoxicity at 48 h of exposure. 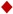 Plant metallic nanoparticles; 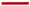 Trendline.

The average size of plant-mediated synthesis of metallic nanoparticles that showed cytotoxicity as fast as 4 h up to 14 days ranged from 20 to 355 nm, although those larger than 100 nm were less commonly synthesised. The average size of the metal plant-synthesised nanoparticles (gold and silver) plotted against LD_50_ or IC_50_ at 24 h exposure (Figure 1) and at 48 h (Figure 2) in vitro are shown.

Both figures show that the cytotoxicity is inversely proportional to size; smaller nanoparticles have smaller LD_50_ or IC_50_ values, which translates to stronger cytotoxicity. This finding is in agreement with a previous study which showed that chemically derived smaller sized gold nanoparticles were highly toxic compared to those of larger sizes, irrespective of the cancer cell types tested [78].

### 2.5. Morphology and Cytotoxicity

**Figure 3:**
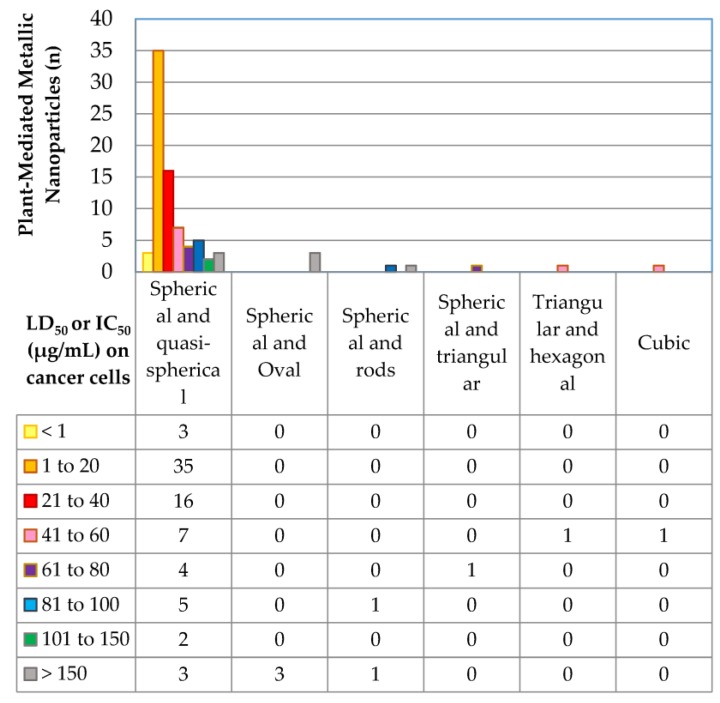
The correlation between plant-mediated syntheses of metallic nanoparticle morphology and cytotoxicity.

Figure 3 shows a comparison of the morphological effect of the plant-mediated syntheses of metallic nanoparticles on their cytotoxicity.

Plant-synthesised metallic nanoparticles vary in shape and can be rods-shaped, spherical, quasi-spherical, oval, triangular, hexagonal, and cubic. In several articles, V-shaped, Y-shaped, and flat plate shaped nanoparticles were described, but those studies were not included for reasons mentioned previously. The plant-mediated syntheses of metallic nanoparticles were predominantly spherical in shape. Those of spherical and quasi-spherical shape had a wide range of cytotoxicity potency, with LD_50_ values ranging from <1 µg/mL to >150 µg/mL. Spherical and quasi-spherical nanoparticles appeared most often, with LD_50_ of 1–20 µg/mL being the mode of the populations, followed by 21–40 and 41–60 µg/mL. Triangular, hexagonal, and rod-shaped nanoparticles were less common and had LD_50_ values of >41 µg/mL (that is, higher than the minimum value of spherical and quasi-spherical shaped nanoparticles).

## 3. Discussion

This systematic review provides a comparison among the studies and more detailed information to explain the complex issues involved in the pursuit of suitable plant-mediated synthesis of metallic nanoparticles for their potential use as anti-cancer agents. The review also provides an overview of LD_50_ and/or cell death of various cancer cells exposed to the synthesised nanoparticles and the extent of agreement or disagreement among the studies regarding their cytotoxicity. Clear documentation of the content of the selected articles is given, which will enable other researchers to assess the validity of the findings. Furthermore, a better understanding of the overall picture of the plant-mediated syntheses of metallic nanoparticle cytotoxicity can be achieved from this review.

Most plant-synthesised metallic nanoparticles discovered to date have a narrow range of doses for their therapeutic index, even when it is regarded as acceptable for oncology indications. Drugs with a narrow therapeutic index are those drugs for which small differences in dose or blood concentration may lead to serious therapeutic failures and/or adverse drug reactions that are life-threatening or result in persistent or significant disability or incapacity [79]. Metallic nanoparticles synthesised by *Abutilon inducum*, *Butea monosperma*, *Gossypium hirsutum*, *Indoneesiella echioides*, and *Melia azedarach* are among the potential anti-cancer agents identified in this review as having an acceptable therapeutic index as a safety marker. Knowing the therapeutic index of a plant-mediated synthesis of metallic nanoparticles can aid in the careful selection of a suitable new entity treatment modality that has a good safety profile for patients or can be used to test subjects before proceeding to animal studies and clinical trials. From the longer-term perspective, this can help reduce the number of expensive research failures in the late stage of clinical trials [80]. However, the therapeutic index results obtained in this review are only preliminary and they could change and be refined as drug development studies progress from in vitro to animal safety to human safety.

Nanoparticles can be taken up into cells through non-specific cellular uptake and via cell functions such as adhesion, cytoskeleton organisation, migration, proliferation, and apoptosis, and these processes may be influenced by nanoparticle shape [81]. Identifying the size and morphology of the plant-mediated syntheses of metallic nanoparticles is important because it will allow for the engineering of nanoparticles with proper shapes and sizes for the maximal accumulation in a malignant tumour. Although nanoparticle size and morphology have significant effects on cytotoxicity potency, other parameters are important as well, such as surface properties, aggregation/agglomeration state, solubility, and surface properties; attached functional groups, surface area, and surface charges also play important roles in influencing the resultant pharmacokinetics and pharmacodynamics of nanoparticles [22,82,83].

The studies analysed in this review included cytotoxicity assays of plant-mediated syntheses of metallic nanoparticles using different cell lines, exposure time lengths, and a wide range of concentrations. This variation made it difficult to make direct comparisons among the available studies. However, the existing data were extracted and the general trends in the pool were evaluated. The results indicate that, ideally designed research studies of plant-mediated syntheses of metallic nanoparticles are lacking. Many of the selected studies compared cytotoxicity of nanoparticles against human cancer cells and non-human normal cells, thus, the therapeutic index could differ when proceeding with clinical trials. Some in vivo studies poorly described exposure conditions such as route of entry and dosing regimen or interval. Additionally, different studies used different standards to measure exposures and outcomes, which might have caused variation in the results displayed. Thus, there is a need for better constructed, suitable, and concrete research that can provide stronger evidence for the efficacy and safety of plant-mediated synthesis of metallic nanoparticles as cytotoxic agents.

## 4. Materials and Methods

### 4.1. Search Strategy

The literature search was executed on full-text electronic databases such as Science Direct, PubMed, and Springer Link. The general search engine Google Scholar and the academic social networking site ResearchGate were also used to obtain research articles. Biosynthesis, plant synthesis, plant-mediated, metallic nanoparticle, cytotoxicity, and anti-cancer were the keywords used for the literature search. A reviewer independently screened the titles and abstracts of the citations and retrieved the relevant articles from the identified full-text electronic journal databases.

**Figure 4:**
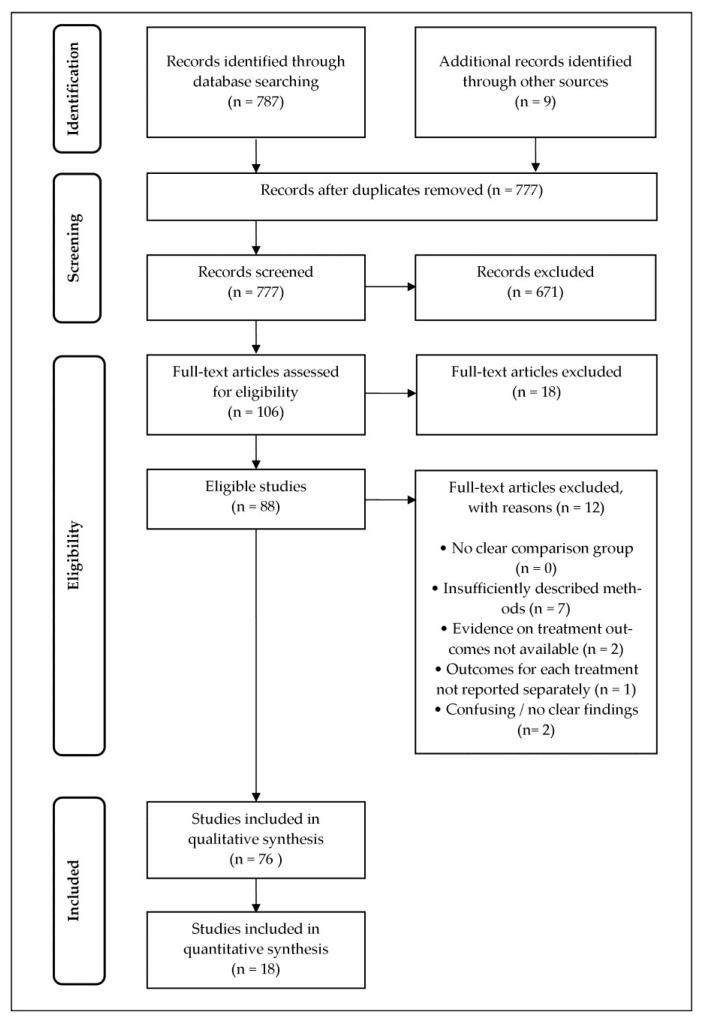
The flow chart of the systematic review information of screening and choosing articles.

With the objective of complementing the database searches, non-automated manual searches of the references within the selected articles were conducted. After the search strategy was applied, two examiners reviewed the screened the research titles and abstracts. The full text of each identified study was retrieved by one author. Documentation of the whole search process was carried out to ensure transparency, replicability, and feasibility to reanalyse (Figure 4).

### 4.2. Study Selection

All types of research (in vitro, in vivo, and ex vivo studies) and reviews that met the inclusion criteria were included in the study regardless of whether the results were positive, negative, or null to diminish selection bias. Preclinical and clinical trials were not included because none had been conducted by the date of this analysis. The inclusion criteria were metallic nanoparticles; gold (Au), silver (Ag); biologically synthesised using plants; recently published studies conducted between 2006 and 2017 (including in-press articles); and all types of studies (in vitro, in vivo, ex vivo, and review). Studies with any of the following criteria were excluded from this study: (1) metallic nanoparticles derived from non-vascular plants (fungus, seaweeds, mushrooms); (2) metallic nanoparticles derived from undefined plants (specific pure compound from an unknown plant used in the biosynthesis); (3) combined synthesis of metallic nanoparticle with chemical or physical methods; (4) combined metallic nanoparticles; (5) studies without available data; and (6) articles that were written in a language other than English.

The titles and abstracts for 796 articles were screened, but only 88 articles were related to the research question. For the 88 potentially eligible articles, the following criteria were established to assess the quality of the study: (1) clearly defined control/comparison group; (2) duration of exposure clearly stated; (3) method conducted at 37 °C to mimic the human body temperature; (4) clearly defined and measured outcomes (median lethal dose (LD_50_), median inhibitory concentration (IC_50_), median growth inhibitory concentration (GI_50_), cell viability, cell death); (5) ethical standards carried out and maintained, if relevant; (6) reliable method used to measure outcomes; (7) multiple measurements of outcome conducted (at least in triplicate); (8) actual data or evidence available on all treatment outcomes; (9) outcomes reported separately for each treatment; (10) clear statement of findings; and (11) appropriate statistical analyses used.

The above study criteria were used with the goal of eliminating attrition biases due to the incomplete reporting of data for each outcome as well as reporting biases from selective reporting of positive outcomes. The following appraisal guidelines were used when developing the study criteria: (1) Preferred Reporting Items for Systematic Review and Meta-Analysis Protocols (PRISMA-P) 2015 checklist [84] and (2) Risk of Bias Assessment Tool for Non-randomised Studies (RoBANS) [85]. Figure 1 shows the methodology used in this study to identify eligible articles.

Data from the selected studies were extracted by one independent author, and that author’s results were counterchecked by two examiners. Descriptions of the evidence were presented in tabular format. The primary outcome measures were LD_50_ and/or IC_50_ for cytotoxicity and therapeutic index for safety. The therapeutic index for the plant-mediated synthesis of metallic nanoparticles was calculated for studies that conducted cytotoxicity testing on both cancer and normal cell types. The therapeutic index is often used to measure the safety of a drug for a particular treatment. It is defined as the ratio of the LD_50_ to the median effective dose (ED_50_) (Equation (1)) [86].

(1) $$\mathrm{Therapeutic}\;\mathrm{Index}\;\left(\mathrm{TI}\right)=\frac{\mathrm{Median}\;\mathrm{Lethal}\;\mathrm{Dose}\;(\mathrm{LD}50)}{\mathrm{Median}\;\mathrm{Effective}\;\mathrm{Dose}\;(\mathrm{EC}50)}$$

LD_50_ is the dose required to kill 50% of the tested population after a specified test duration, and ED_50_ is the dose required to achieve the desired outcome or response in 50% of the tested population. Based on the availability of a reasonable therapeutic index value, the potential plant-mediated syntheses of metallic nanoparticles with the most promise as anticancer agents were selected. The correlation between the average size/morphology of plant-synthesised metallic nanoparticles and cytotoxicity potency was also analysed.

## 5. Conclusions

With respect to the above-mentioned results and discussion, it is concluded that silver plant-mediated nanoparticles have higher cytotoxicity compared to gold, irrespective of the cell types used. The size and shape determined the resultant cytotoxicity of the plant-mediated synthesis of metallic nanoparticles. Most of the nanoparticles have a narrow therapeutic index despite the acceptance of oncology indications. Metallic nanoparticles synthesised from *Abutilon inducum*, *Butea monosperma*, *Gossypium hirsutum*, *Indoneesiella echioides*, and *Melia azedarach* are among the potential anti-cancer agents with an acceptable therapeutic index as the safety marker. Future studies should focus on the pharmacological/toxicological and pharmacokinetic/toxicokinetic actions of the potential anti-cancer agents.

## Figures and Tables

**Table 1:** The cytotoxicity of the plant-mediated syntheses of metallic nanoparticles on cancer cells (in vitro).

Cancer Cell Line		LD_50_ or IC_50_	Cell Death	Exposure Duration	Response Relationship	Metallic Nanoparticle	Plant Used	Plant Part	Mechanism of Action	Ref.	Year
Lung	A549	NA	Complete cell death at 10 µg/mL	4 h	Dose-dependent	Ag	*Cassia auriculata*	Leaf	Not studied	[14]	2015
		10 µg/mL	Complete cell death 30 µg/mL	4 h	Dose-dependent	Au	*Cassia auriculata*	Leaf	Not studied		
		13.5 µg/mL	>80% cell death at >40 µg/mL	4 h	Dose-dependent	Ag	*Jatropha gossypifolia*	Stem	Not studied	[15]	2014
		19.5 µg/mL	>80% cell death at >40 µg/mL	4 h	Dose-dependent	Ag	*Jatropha curcus*	Stem	Not studied		
		20 µg/mL	NA	24 h	Dose-dependent	Ag	*Euphorbia nivulia*	Latex	Apoptosis	[16]	2011
		28.125 µg/mL	NA	24 h	Dose-dependent	Ag	*Bauhinia tomentosa* (Kanchini)	Leaf	Not studied	[12]	2015
		28.37 µg/mL	NA	24 h	Dose-dependent	Au	*Nigella sativa*	Essential oil from seed	Not studied	[17]	2016
		53.2 µg/mL	NA	24 h	Time and Dose-dependent	Ag	*Acorous calamus*	Rhizome	Apoptosis	[18]	2014
		80 µg/mL	NA	24 h	Dose-dependent	Ag	*Rosa damascena*	Flower petal	Not studied	[19]	2014
		100 µg/mL	<20% at 500 µg/mL	36 h	Dose-dependent	Ag	*Origanum vulgare*	Leaf	Reduce cell proliferation, increase ROS, DNA fragmentation, apoptosis	[20]	2013
		30 µg/mL	NA	48 h	Dose-dependent	Ag	*Indoneesiella echioides*	Leaf	Not studied	[5]	2016
		32.1 µg/mL	NA	48 h	Time and Dose-dependent	Ag	*Acorous calamus*	Rhizome	Apoptosis	[18]	2014
		40 µg/mL	NA	48 h	Dose-dependent	Ag	*Gossypium hirsutum*	Leaf	Inhibit cell proliferation, induce loss of cell membrane integrity, apoptosis	[21]	2014
		NA	80.2% at 200 nM	48 h	Dose-dependent	Au	*Illicum verum*(Star Anise)	Deseeded pod	Apoptosis	[22]	2015
		NA	<10% cell death in 0.01–20 µM	48 h	Non-cytotoxic	Au	*Genipa americana*	Fruit	Not studied	[23]	2016
Liver	HepG2	6 µg/mL	NA	24 h	Time and Dose-dependent	Au	*Cajanus cajan*	Seed coat	Apoptosis	[24]	2014
		NA	≈80% cell death at 2 µg/mL	48 h							
		49.5 µg/mL	>80% cell death at 100 µg/mL	48 h	Dose-dependent	Ag	*Oocimum kilimandscharicum*	Stem	Not studied	[6]	2015
		NA (GI_50_ = 93.75 µg/mL)	16.39% cell death at 1 mg/mL	48 h	Dose-dependent	Ag	*Morinda pubescens*	Leaf	Not studied	[25]	2013
	Hep3B	150 µg/mL	20% cell death at 200 µg/mL	24 h	Dose-dependent	Au	*Rhus chinensis*	Plant gall	Not studied	[26]	2016
Colorectal	HCT15	8 µg/mL	NA	24 h	Time and Dose-dependent	Ag	*Vitex negundo*	Leaf	Inhibit proliferation, cell cycle arrest, apoptosis	[27]	2014
		4 µg/mL	NA	48 h							2014
		20 µg/mL	NA	48 h	Dose-dependent	Ag	*Vitex negundo*	Leaf	Apoptosis, cell cycle arrest	[28]	2013
	HCT116	100 µg/mL	>60% cell death at 400 µg/mL	24 h	Dose-dependent	Ag	*Commelina nudiflora*	Not stated	Apoptosis	[29]	2016
		200 µg/mL	>70% cell death at 400 µg/mL	24 h	Dose-dependent	Au	*Commelina nudiflora*	Not stated	Apoptosis		
	HT29	30 µg/mL	NA	12 h	Time and Dose-dependent	Ag	*Couroupita guainensis*	Leaf	Not studied	[30]	2016
		6 µg/mL	NA	24 h	Time and Dose-dependent	Ag	*Vitex negundo*	Leaf	Inhibit proliferation, cell cycle arrest, apoptosis	[27]	2014
		15 µg/mL	NA	24 h	Time and Dose-dependent	Ag	*Terminalia chebula*	Fruit	Not studied	[10]	2016
		23.44 µg/mL	NA	24 h	Dose-dependent	Ag	*Catharanthus roseus*	Leaf	Not studied	[31]	2015
		25 µg/mL	>75% cell death at 40 µg/mL	24 h	Time and Dose-dependent	Ag	*Couroupita guainensis*	Leaf	Not studied	[30]	2016
		210 µg/mL	NA	24 h	Dose-dependent	Au	*Abutilon indicum*	Leaf	DNA damage, arrest cell cycle, apoptosis	[32]	2016
		2 µg/mL	NA	48 h	Time and Dose-dependent	Ag	*Vitex negundo*	Leaf	Inhibit proliferation, cell cycle arrest, apoptosis	[27]	2014
		10 µg/mL	NA	48 h	Time and Dose-dependent	Ag	*Terminalia chebula*	Fruit	Not studied	[10]	2016
		20 µg/mL	>75% cell death at 40 µg/mL	48 h	Time and Dose-dependent	Ag	*Couroupita guainensis*	Leaf	Not studied	[30]	2016
		39.06 µg/mL	NA	48 h	Dose-dependent	Ag	*Catharanthus roseus*	Leaf	Not studied	[31]	2015
		180 µg/mL	NA	48 h	Dose-dependent	Au	*Abutilon indicum*	Leaf	DNA damage, arrest cell cycle, apoptosis	[32]	2016
		46.88 µg/mL	NA	72 h	Dose-dependent	Ag	*Catharanthus roseus*	Leaf	Not studied	[31]	2015
	Caco-2	10 µM	≈80% cell death at 50 µM	48 h	Dose-dependent	Ag	*Eclipta alba*	Leaf	Not studied	[33]	2015
	Colo 205	4 µg/mL	NA	24 h	Time and Dose-dependent	Ag	*Abutilon inducum*	Leaf	DNA damage, arrest cell cycle, apoptosis	[32]	2016
		5.5 µg/mL	NA	24 h	Time and Dose-dependent	Ag	*Plumeria alba*	Flower petal	Apoptosis	[7]	2015
		3 µg/mL	NA	48 h	Time and Dose-dependent	Ag	*Abutilon inducum*	Leaf	DNA damage, arrest cell cycle, apoptosis	[34]	2015
		4.5 µg/mL	NA	48 h	Time and Dose-dependent	Ag	*Plumeria alba*	Flower petal	Apoptosis	[7]	2015
	C26 (murine)	NA	<20% at 6 µg/mL and >80% at 8 µg/mL	24 h	Dose-dependent	Ag	*Azadirachta indica*	Leaf	Not studied	[35]	2015
Stomach	AGS	NA	<30% cell death in 3.125 to 200 µg/mL for all duration	8, 16, 24 h	Minimally Dose-dependent	Au	*Tribulus terrestris*	Fruit	Apoptosis	[36]	2016
	MKN 28	150 µg/mL	80% at 200 µg/mL	24 h	Dose-dependent	Au	*Rhus chinensis*	Plant gall	Not studied	[26]	2016
Breast	MCF7	0.024 µg/mL	NA	24 h	Dose-dependent	Ag	*Oleo europaea*	Leaf	Not studied	[37]	2014
		4.91 µg/mL	NA	24 h	Dose-dependent	Ag	*Potentilla fulgens*	Root	Apoptosis	[11]	2015
		5 µg/mL	NA	24 h	Dose-dependent	Ag	*Dendrophthoe falcata*	Leaf	Not studied	[38]	2014
		67 µg/mL	NA	24 h	Time and Dose-dependent	Ag	*Piper longum*	Fruit	Not studied	[39]	2014
		217 µg/mL	NA	24 h	Dose-dependent	Ag	*Adenium abesum*	Leaf	DNA damage, autophagy via increased ROS, apoptosis	[40]	2016
		7.19 µg/mL	NA	24 h	Dose-dependent	Ag	*Cassia fistula*	Flower	Apoptosis	[41]	2015
		<8 µg/mL	NA	24 h	Time and Dose-dependent	Au	*Musa paradisiaca*	Pectin	Apoptosis	[42]	2016
		20 µg/mL	NA	24 h	Dose-dependent	Ag	*Datura inoxia*	Leaf	Growth suppression, cell cycle arrest, DNA synthesis reduction, apoptosis	[43]	2014
		20 µg/mL	Complete cell death at 50 µg/mL	24 h	Dose-dependent	Ag	*Sesbania grandiflora*	Leaf	DNA damage, oxidative stress induction, apoptosis	[44]	2013
		30 µg/mL	NA	24 h	Dose-dependent	Ag	*Solnum trilobatum*	Fruit (unripe)	Apoptosis	[45]	2015
		30.5 µg/mL	Complete cell inhibition at 100 µg/mL	24 h	Dose-dependent	Ag	*Coriandrum sativum*	Leaf	Not studied	[46]	2016
		42.5 µg/mL	98% cell inhibition at 100 µg/mL	24 h	Dose-dependent	Ag	*Alternanthera tenella*	Leaf	Not studied	[47]	2016
		50 µg/mL	NA	24 h	Time and Dose-dependent	Ag	*Annona squamosa*	Leaf	Apoptosis	[48]	2012
		NA	≈80% cell death at 2 µg/mL	24 h	Inversely Dose-dependent	Au	*Camellia sinensis, Coriandrum sativum*, *Mentha arvensis*, *Phyllanthus amarus*, *Artabotrys hexapetalus*, *Mimusops elengi*, *Syzygium aromaticum*, *C. sinensis*	Leaf	Not studied	[49]	2015
		8 µg/mL	NA	48 h	Time and Dose-dependent	Au	*Musa paradisiaca*	Pectin	Apoptosis	[42]	2016
		30 µg/mL	NA	24 h	Time and Dose-dependent	Ag	*Annona squamosa*	Leaf	Apoptosis	[48]	2012
		10 µg/mL	NA	48 h	Time and Dose-dependent	Ag	*Malus domestica*	Fruit	Not studied	[50]	2014
		31.2 µg/mL	NA	48 h	Dose-dependent	Ag	*Melia dubia*	Leaf	Not studied	[51]	2014
		51 µg/mL	NA	48 h	Time and Dose-dependent	Ag	*Piper longum*	Fruit	Not studied	[39]	2014
		NA (GI_50_ = 257.8 µg/mL)	NA	48 h	Dose-dependent	Au	*Antigonon leptopus*	Leaf	Not studied	[52]	2015
		0.455 µg/mL	NA	72 h	Dose-dependent	Au	*Nelsonia canescens*	Leaf	Not studied	[53]	2016
		NA	<60% cell death at 5 µg/mL and above	72 h	Dose-dependent	Ag	*Acacia*	Lignin from wood	Not studied	[54]	2016
		100 µg/mL	>80% cell death at 500 µg/mL	120 h	Dose-dependent	Ag	*Origanum heracleoticum*	Leaf	Not studied	[55]	2015
	MDA-MB-231	<10 µg/mL	Complete cell death at 10 µg/mL	4 h	Dose-dependent	Ag	*Cassia auriculata*	Leaf	Not studied	[14]	2015
		10 µg/mL	Complete cell death at 30 µg/mL	4 h	Dose-dependent	Au	*Cassia auriculata*	Leaf	Not studied		
		<2 µg/mL	NA	24 h	Time and Dose-dependent	Au	*Musa paradisiaca*	Pectin	Apoptosis	[42]	2016
		2 µg/mL	NA	48 h	Dose-dependent	Au	*Musa paradisiaca*	Pectin	Apoptosis	[42]	2016
Cervix	HeLa	51 µg/mL	88% cell death at 300 µg/mL	3 h	Dose-dependent	Ag	*Iresine herbstii*	Leaf	Not studied	[9]	2012
		20 µg/mL	NA	24 h	Dose-dependent	Au	*Podophyllum hexandrum*	Leaf	DNA damage, oxidative stress induction, apoptosis	[56]	2014
		87.32 µg/mL	NA	24 h	Dose-dependent	Ag	*Nothapodytes nimmoniana*	Fruit (ripe)	Not studied	[57]	2016
		92.48 µg/mL	NA	24 h	Time and Dose-dependent	Ag	*Acorous calamus*	Rhizome	Apoptosis	[18]	2014
		28 µg/mL	NA	48 h	Dose-dependent	Ag	*Euphorbia antiquorum*	Latex	ROS	[58]	2016
		47.77 µg/mL	NA	48 h	Dose-dependent	Au	*Albizia amara*	Leaf	Not studied	[59]	2017
		62.5 µg/mL	Almost 100% cell death at 1000 µg/mL	48 h	Dose-dependent	Au	*Punica granatum*	Fruit	Not studied	[60]	2014
		69.44 µg/mL	NA	48 h	Time and Dose-dependent	Ag	*Acorous calamus*	Rhizome	Apoptosis	[18]	2014
		NA (GI_50_ = 34.5 µg/mL)	NA	48 h	Dose-dependent	Ag	*Cymodocea serrulata*	Whole plant	Not studied	[61]	2015
		300 µg/mL	NA	48–72 h	Dose-dependent	Ag	*Melia azedarach*	Leaf	Apoptosis	[62]	2012
Brain	U87	8.23 µg/mL	NA	24 h	Dose-dependent	Ag	*Potentilla fulgens*	Root	Apoptosis	[11]	2015
		1.5 ng/mL	NA	48 h	Dose-dependent	Au	*Hibiscus sabdariffa*	Leaf and stem (optimal: leaf)	GADPH enzyme degradation	[8]	2016
Blood	HL-60	2 mmol/L	NA	6 h	Time and Dose-dependent	Ag	*Eucalyptus chapmania*	Leaf	Not studied	[63]	2013
		1 mmol/L	NA	24 h							
		5.14 μM	NA	120 h	Dose-dependent	Au	*Couroupita guianensis*	Flower	Apoptosis	[64]	2013
	Jurkat	13.64 µg/mL	NA	24 h	Dose-dependent	Ag	*Abelmoschus esculentus*	Pulp	ROS and NO production	[65]	2015
		27.35 µg/mL	NA	24 h	Dose-dependent	Ag	*Catharanthus roseus*	Leaf	Not studied	[31]	2015
		39.06 µg/mL	NA	48 h							
		46.88 µg/mL	NA	72 h	Dose-dependent						
	KG-1A	11.47 µg/mL	NA	24 h	Dose-dependent	Ag	*Butea monosperma*	Bark	Apoptosis	[66]	2015
Bone	MG63	150 µg/mL	80% at 200 µg/mL	24 h	Dose-dependent	Au	*Rhus chinensis*	Plant gall	Not studied	[26]	2016
		75.5 ± 2.4 µg/mL	NA	48 h	Dose-dependent	Ag	*Ficus benghalensis*	Bark	Not studied	[67]	2016
		81.8 ± 2.6µg/mL	NA	48 h	Dose-dependent	Ag	*Azadirachta indica*	Bark	Not studied		
Connective tissue	HT1080	NA	<5% cell death at up to 200µM	6–24 h	Non-cytotoxic	Au	*Dysosma pleiantha*	Rhizome	Cell migration inhibition via Rac1 mediated actin polymerization pathway	[68]	2013
Prostate	LNCap-FGC	<10 µg/mL	Complete cell death at 10 µg/mL	4 h	Dose-dependent	Ag	*Cassia auriculata*	Leaf	Not studied	[14]	2015
		10 µg/mL	Complete cell death at 30 µg/mL	4 h	Dose-dependent	Au	*Cassia auriculata*	Leaf	Not studied		
Skin	A375	NA	>75% cell death at 5 µg/mL	72 h	Dose-dependent	Ag	Acacia	Lignin from wood	Not studied	[54]	2016
Throat	Hep-2	20 µg/mL	Complete cell death at 40 µg/mL	24 h	Dose-dependent	Ag	*Phyllanthus emblica*	Fruit	Cell proliferation reduction, ROS production, DNA fragmentation, apoptosis	[69]	2013
		31.25 µg/mL	94.02% at 500 µg/mL	24 h	Dose-dependent	Ag	*Piper longum*	Leaf	ROS	[70]	2012
Dalton’sAscitesLymphoma (DAL)		NA	65.61% cell death at 150 µg/mL	24 h	Dose-dependent	Ag	*Plumbago zeylanica*	Bark	Not studied	[71]	2016
		NA	61.56% cell death at 150 µg/mL	24 h	Dose-dependent	Au					

NA = Not Available.

**Table 2:** The cytotoxicity of plant-mediated syntheses of metallic nanoparticle on normal cells (in vitro).

Healthy Cell Line		LD_50_or IC_50_	Cell Death	Exposure Duration	Response Relationship	Metallic Nanoparticle	Plant Used	Plant Part	Mechanism of Action	Ref.	Year
Blood	Lymphocyte	NA	<20% cell death at 6 µg/mL	24 h	Dose-dependent	Ag	*Potentilla fulgens*	Root	Apoptosis	[11]	2015
	PBMC	43.18 µg/mL	NA	24 h	Dose-dependent	Ag	*Butea monosperma*	Bark	Apoptosis	[66]	2015
		113.25 μM	NA	120 h	Dose-dependent	Au	*Couroupita guianensis*	Flower	Apoptosis	[64]	2013
		NA	<20% cell death in 0.008 to 0.04 µg/mL	24 h	NA	Ag	*Oleo europaea*	Leaf	Not studied	[37]	2014
Breast	HBL100	80 µg/mL	NA	24 h	Time and Dose-dependent	Ag	*Annona squamosa*	Leaf	Apoptosis	[48]	2012
		60 µg/mL	NA	48 h	Dose-dependent	Ag	*Indoneesiella echioides*	Leaf	Not studied	[5]	2016
		60 µg/mL	NA	48 h	Time and Dose-dependent	Ag	*Annona squamosa*	Leaf	Apoptosis	[48]	2012
		80 µg/mL	NA	48 h	Dose-dependent	Ag	*Gossypium hirsutum*	Leaf	Inhibit cell proliferation, induce loss of cell membrane integrity, apoptosis	[21]	2014
		750 µg/mL	NA	48–72 h	Dose-dependent	Ag	*Melia azedarach*	Leaf	Apoptosis	[62]	2012
Colon	Normal colon	50 µg/mL	NA	12 h	Time and Dose-dependent	Ag	*Couroupita guainensis*	Leaf	Not studied	[30]	2016
		40 µg/mL	NA	24 h	Time and Dose-dependent with saturation effect						
		40 µg/mL	NA	48 h							
Skin	HaCaT	1000 µg/mL	NA	24 h	Time and Dose-dependent	Au	*Abutilon indicum*	Leaf	DNA damage, arrest cell cycle, apoptosis	[32]	2016
		900 µg/mL	NA	48 h							
		NA	<2% at <6 µg/mL and >75%% at >8 µg/mL	24 h	Dose-dependent	Ag	*Azadirachta indica*	Leaf	Not studied	[35]	2015
	HSFs	NA	>50% cell death in 16–80 µg/mL	24 h	Dose-dependent with saturation effect	Ag	*Theobroma cacao*	Beans (S4H formula)	Not studied	[72]	2016
		NA	>50% cell death at 80 µg/mL	24 h	Dose-dependent			Beans (S3H formula)			
		NA	>50% cell death at 16 µg/mL	72 h	Dose-dependent			Beans (S3H formula)			
		NA	>50% cell death in 16–80 µg/mL	72 h	Dose-dependent with saturation effect			Beans (S4H formula)			
Foetallung	W1-38	NA	<20% cell death in 10 to 160 µg/mL	24 h	Non cytotoxic	Au	*Mangifera indica*	Peel	NA	[73]	2014
Kidney	Embryonic human kidney (293)	NA (LD_20_ = 2 ng/mL)	NA	48 h	Dose-dependent	Au	*Hibiscus sabdariffa*	Leaf and stem (leaf gives optimal yield)	GADPH enzyme degradation	[8]	2016
	Madin Darby canine kidney (MDCK)	100 µg/mL	NA	24 h	Time and Dose-dependent	Ag	*Abutilon inducum*	Leaf	DNA damage, arrest cell cycle, apoptosis	[32]	2016
		75 µg/mL	NA	48 h							
	African green monkey kidney (Vero)	20 µg/mL	NA	24 h	Time and Dose-dependent	Ag	*Terminalia chebula*	Fruit	Not studied	[10]	2016
		66.34 µg/mL	NA	24 h	Dose-dependent	Ag	*Cassia fistula*	Flower	Apoptosis	[41]	2015
		246 µg/mL	NA	24 h	Dose-dependent	Au	*Cajanus cajan*	Seed coat	Apoptosis	[24]	2014
		NA	72.8% cell inhibition at 20 µg/mL	24 h	Dose-dependent	Ag	*Datura inoxia*	Leaf	Growth suppression, cell cycle arrest, DNA synthesis reduction, apoptosis	[43]	2014
		30 µg/mL	NA	48 h	Time and Dose-dependent	Ag	*Terminalia chebula*	Fruit	Not studied	[10]	2016
		72.28 µg/mL	NA	48 h	Dose-dependent	Au	*Albizia amara*	Leaf	Not studied	[59]	2017
		500 µg/mL	NA	48 h	Dose-dependent	Ag	*Melia dubia*	Leaf	Not studied	[51]	2014
		NA (GI_50_ = 61.24 µg/mL)	NA	48 h	Dose-dependent	Ag	*Cymodocea serrulata*	Whole plant	Not studied	[61]	2015
		NA	<10% cell death in 0.01–20 µM	48 h	Non-cytotoxic	Au	*Genipa americana*	Fruit	Not studied	[23]	2016
		18.79 µg/mL	NA	72 h	Dose-dependent	Ag	*Aegiceras corniculatum*	Leaf	Not studied	[74]	2017
	CV-1	NA	<20% cell death in 10 to 160 µg/mL	24 h	Non cytotoxic	Au	*Mangifera indica*	Peel	NA	[73]	2014
Adipose	3T3-L1 (murine)	NA (LD_20_ = 10 µg/mL)	NA	24 h	Dose-dependent with saturation effect	Au	*Torreya nucifera*	Leaf	Not studied	[75]	2013
		NA	>20% cell death at 0.1 ng/mL and above	24 h		Au	*Cinnamomum japonicum*	Leaf			
		NA (LD_20_ = 100 ng/mL)	NA	24 h		Au	*Nerium indicum*	Leaf			
Mousefibroblast	L929	NA	<20% cell death up to 250 µg/mL	24 h	Non cytotoxic	Ag	*Nyctanthes arbortristis*	Flower	NA	[76]	2015

NA = Not Available.

**Table 3:** The cytotoxicity of plant-mediated syntheses of metallic nanoparticles on animals (in vivo).

Subject	IC_50_	Toxicity	Exposure Duration	Response Relationship	Metallic Nanoparticle	Plant	Plant Part	Ref.	Year
Adult Zebrafish (*Danio rerio*)	142.2 ng/mL	Aggressive behaviours and jerky movements after 6 h of treatment prior to mortality; 100% mortality at 331.8 ng/mL (48 h); 100% mortality at 284.4 ng/mL (96 h)	96 h (Single dose)	Dose-dependent	Ag	*Malva crispa*	Leaf	[77]	2016
	Dose used = 71.1 ng/mL	Gill tissue cell membrane damage; irregular cell outlines and complete disruption of gill cells; evidence of genotoxicity in peripheral blood erythrocytes for AgNP exposed zebrafish	14 days (given once daily)	NA					
